# 4-Meth­oxy-*N*-[(*E*)-(5-nitro­thio­phen-2-yl)methyl­idene]aniline

**DOI:** 10.1107/S1600536812034344

**Published:** 2012-09-29

**Authors:** Tufan Akbal, Erbil Ağar, Sümeyye Gümüş, Ahmet Erdönmez

**Affiliations:** a Department of Physics, Arts and Sciences Faculty, Ondokuz Mayıs University, 55139 Samsun, Turkey; b Department of Chemistry, Arts and Sciences Faculty, Ondokuz Mayıs University, 55139 Samsun, Turkey

## Abstract

The title mol­ecule, C_12_H_10_N_2_O_3_S, is nonplanar with an inter­planar angle of 33.44 (7)° between the benzene and thio­phene rings. In the crystal there exist only weak inter­molecular C—H⋯O inter­actions, π–π inter­actions between the benzene rings [centroid–centroid distance = 3.7465 (14) Å] and *X*—*Y*⋯π inter­actions to the thio­phene and benzene rings [N⋯centroid distances = 3.718 (3) and 3.355 (3) Å, respectively]. Inter­molecular C—H⋯O inter­actions link the mol­ecules into chains parallel to the *a* axis.

## Related literature

For the biological properties of Schiff bases, see Layer (1963 ▶); Ingold (1969 ▶); Barton & Ollis (1979 ▶). For the application of Schiff bases in industry, see Taggi *et al.* (2002 ▶). For related structures, see Ceylan *et al.* (2011 ▶); Özdemir & Işık (2012 ▶).
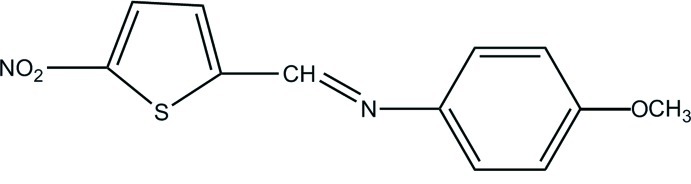


## Experimental

### 

#### Crystal data

C_12_H_10_N_2_O_3_S
*M* *_r_* = 262.28
Monoclinic, 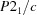

*a* = 12.5641 (7) Å
*b* = 13.1441 (5) Å
*c* = 7.7896 (4) Å
β = 106.012 (4)°
*V* = 1236.50 (10) Å^3^
*Z* = 4
Mo *K*α radiation
μ = 0.26 mm^−1^
*T* = 296 K
0.69 × 0.51 × 0.28 mm

#### Data collection

Stoe IPDS 2 diffractometer
Absorption correction: integration (*X-RED32*; Stoe & Cie, 2002 ▶) *T*_min_ = 0.873, *T*_max_ = 0.938
20097 measured reflections
2841 independent reflections
2076 reflections with *I* > 2σ(*I*)
*R*_int_ = 0.053

#### Refinement

*R*[*F* ^2^ > 2σ(*F* ^2^)] = 0.047
*wR*(*F* ^2^) = 0.125
*S* = 1.15
2841 reflections
164 parameters
H-atom parameters constrained
Δρ_max_ = 0.29 e Å^−3^
Δρ_min_ = −0.22 e Å^−3^

### 

Data collection: *X-AREA* (Stoe & Cie, 2002 ▶); cell refinement: *X-AREA*; data reduction: *X-RED32* (Stoe & Cie, 2002 ▶); program(s) used to solve structure: *WinGX* (Farrugia, 1997 ▶) and *SHELXS97* (Sheldrick, 2008 ▶); program(s) used to refine structure: *SHELXL97* (Sheldrick, 2008 ▶); molecular graphics: *ORTEP-3 for Windows* (Farrugia, 1997 ▶); software used to prepare material for publication: *WinGX* (Farrugia, 1999 ▶) and *PLATON* (Spek, 2009 ▶).

## Supplementary Material

Crystal structure: contains datablock(s) I, global. DOI: 10.1107/S1600536812034344/fb2260sup1.cif

Supplementary material file. DOI: 10.1107/S1600536812034344/fb2260Isup2.mol

Structure factors: contains datablock(s) I. DOI: 10.1107/S1600536812034344/fb2260Isup3.hkl

Supplementary material file. DOI: 10.1107/S1600536812034344/fb2260Isup4.cml

Additional supplementary materials:  crystallographic information; 3D view; checkCIF report

## Figures and Tables

**Table 1:** Hydrogen-bond geometry (Å, °)

*D*—H⋯*A*	*D*—H	H⋯*A*	*D*⋯*A*	*D*—H⋯*A*
C8—H8⋯O2^i^	0.93	2.48	3.292 (3)	146
C9—H9⋯O3^ii^	0.93	2.40	3.273 (3)	156

Symmetry codes: (i) 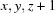; (ii) 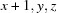.

**Fig. 1.**
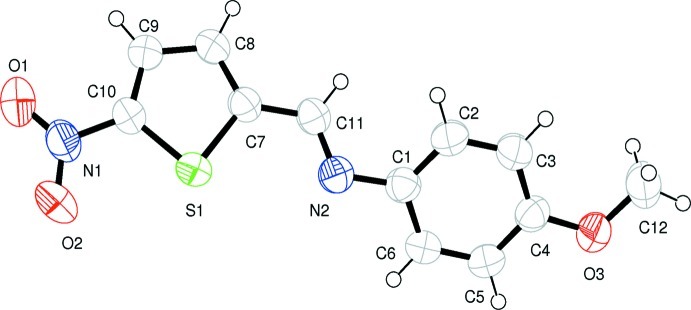
The title molecule with the atom-numbering scheme. The displacement ellipsoids are drawn at the 30% probability level.

**Fig. 2.**
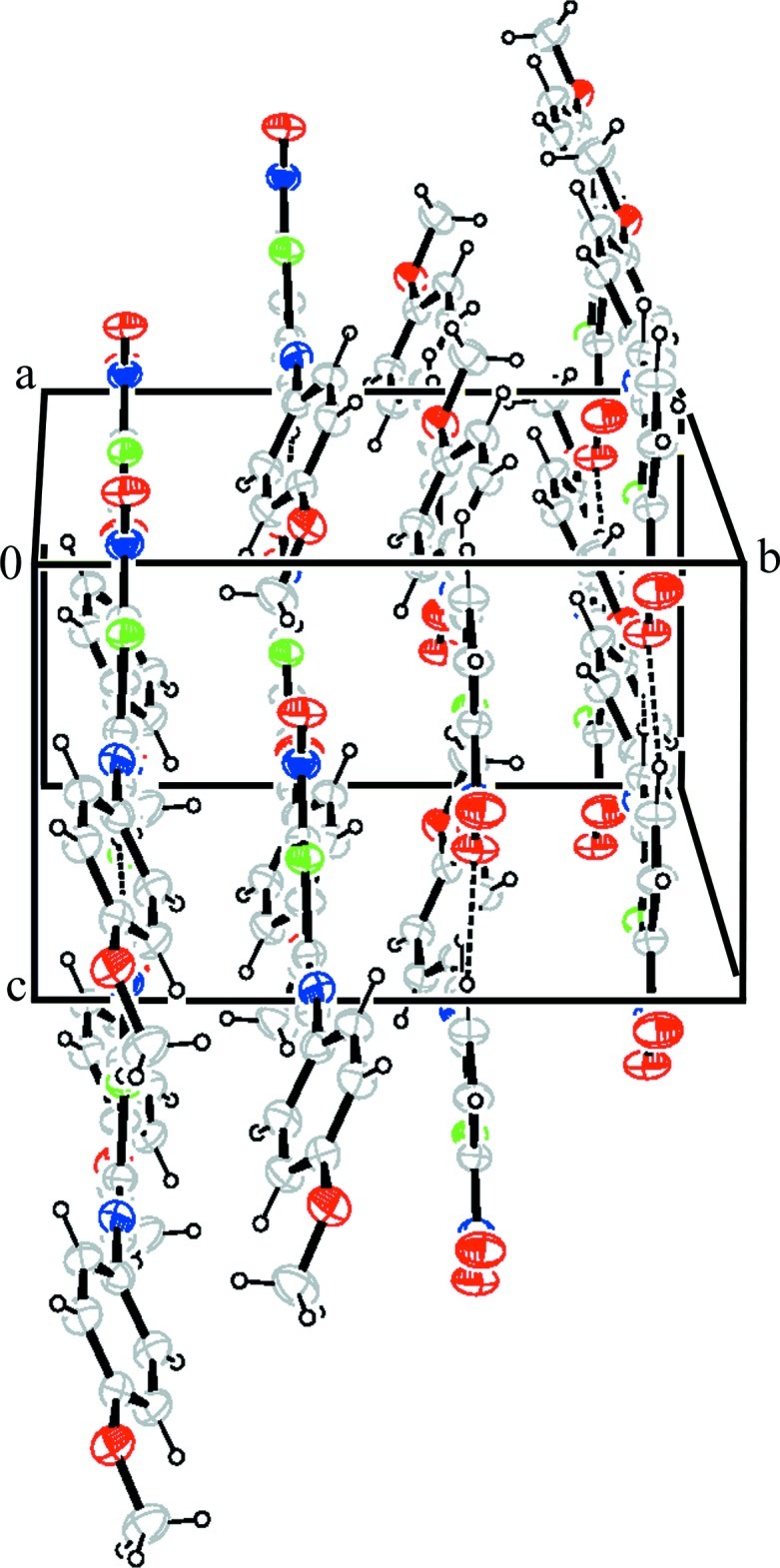
A view of the crystal packing of the title compound.

## supplementary crystallographic information

### Comment

The Schiff bases, *i. e.* the compounds having a double C═N bond, are used as starting materials in the synthesis of important drugs, such as antibiotics and antiallergic, antiphlogistic, and antitumor substances (Layer, 1963; Ingold, 1969; Barton & Ollis, 1979). On the industrial scale, they have a wide range of applications, such as dyes and pigments (Taggi *et al.*, 2002).

The title molecule is shown in Fig. 1. The molecule is non-planar, with an interplanar angle of 33.44 (7)° between the benzene and the substituted thiophene rings. The length of the C11═N2 double bond is 1.268 (3) Å. This value agrees well with the analogous bond reported elsewhere (Ceylan *et al.* 2011; Özdemir Tari & Işık, 2012). In the crystal (Fig. 2), two different C—H···O intermolecular interactions (Table 1) generate chains of molecules extending along the *a* axis. The distance 3.7465 (14) Å between the centroids of the neighbouring benzene rings related by the symmetry operation 1-*x*, -*y*, 1-*z* indicates a π-electron—π-electron ring interaction. Intermolecular X—Y···π-electron ring interactions are also present in the crystal structure (N1—O1···*Cg*1^i^=3.718 (3) Å and N1—O2···*Cg2*^ii^=3.355 (3) Å where *Cg*1 and *Cg*2 are the centroids of the rings C7—C10/S1 (substituted thiophene) and C1—C6 (benzene), respectively, and the symmetry codes i and ii correspond to 2-*x*, -*y*, -*z* and *x*, 1/2-*y*, -1/2+*z*, respectively.

### Experimental

The title compound was prepared under reflux at room temperature of a mixture of two solutions: One contained 5-nitro-2-thiophene-carboxaldehyde (0.016 g, 0.100 mmol) in 20 ml of absolute ethanol, while the other 4-methoxyaniline (0.012 g, 0.100 mmol) in 20 ml of absolute ethanol. The reaction mixture was stirred for 1 h under reflux. Yellow, transparent crystals were obtained by slow evaporation from ethanol solution at room temperature (yield 68 wt. %; m.p. 414–417 K). The average size of the prismatic crystals was about 0.12 × 0.45 × 0.80 mm.

### Refinement

All the hydrogen atoms appeared in the difference electron density map, nevertheless, they were situated into the idealized positions and refined in the riding atom formalism. The applied constraints: C_methyl_—H_methyl_=0.96, C_aryl_—H_aryl_=0.93 Å. *U*_iso_(H_methyl_) = 1.5*U*_eq_(C_methyl_), *U*_iso_(H_aryl_) = 1.2*U*_eq_(C_aryl_).

### Crystal data

**Table:**

C_12_H_10_N_2_O_3_S	*F*(000) = 544
*M**_r_* = 262.28	*D*_x_ = 1.409 Mg m^−^^3^
Monoclinic, *P*2_1_/*c*	Melting point = 414–417 K
Hall symbol: -P 2ybc	Mo *K*α radiation, λ = 0.71073 Å
*a* = 12.5641 (7) Å	Cell parameters from 20097 reflections
*b* = 13.1441 (5) Å	θ = 2.3–27.6°
*c* = 7.7896 (4) Å	µ = 0.26 mm^−^^1^
β = 106.012 (4)°	*T* = 296 K
*V* = 1236.50 (10) Å^3^	Prism, yellow
*Z* = 4	0.69 × 0.51 × 0.28 mm

### Data collection

**Table:**

Stoe IPDS 2 diffractometer	2841 independent reflections
Radiation source: fine-focus sealed tube	2076 reflections with *I* > 2σ(*I*)
Graphite monochromator	*R*_int_ = 0.053
w–scan rotation	θ_max_ = 27.6°, θ_min_ = 2.3°
Absorption correction: integration (*X-RED32*; Stoe & Cie, 2002)	*h* = −16→16
*T*_min_ = 0.873, *T*_max_ = 0.938	*k* = −17→17
20097 measured reflections	*l* = −10→9

### Refinement

**Table:**

Refinement on *F*^2^	Secondary atom site location: difference Fourier map
Least-squares matrix: full	Hydrogen site location: difference Fourier map
*R*[*F*^2^ > 2σ(*F*^2^)] = 0.047	H-atom parameters constrained
*wR*(*F*^2^) = 0.125	*w* = 1/[σ^2^(*F*_o_^2^) + (0.0447*P*)^2^ + 0.3031*P*] where *P* = (*F*_o_^2^ + 2*F*_c_^2^)/3
*S* = 1.15	(Δ/σ)_max_ < 0.001
2841 reflections	Δρ_max_ = 0.29 e Å^−^^3^
164 parameters	Δρ_min_ = −0.22 e Å^−^^3^
0 restraints	Extinction correction: *SHELXL97* (Sheldrick, 2008)
39 constraints	Extinction coefficient: 0
Primary atom site location: structure-invariant direct methods	

### Special details

**Table:**

Geometry. All e.s.d.'s (except the e.s.d. in the dihedral angle between two l.s. planes) are estimated using the full covariance matrix. The cell e.s.d.'s are taken into account individually in the estimation of e.s.d.'s in distances, angles and torsion angles; correlations between e.s.d.'s in cell parameters are only used when they are defined by crystal symmetry. An approximate (isotropic) treatment of cell e.s.d.'s is used for estimating e.s.d.'s involving l.s. planes.
Refinement. Refinement of *F*^2^ against ALL reflections. The weighted *R*-factor *wR* and goodness of fit *S* are based on *F*^2^, conventional *R*-factors *R* are based on *F*, with *F* set to zero for negative *F*^2^. The threshold expression of *F*^2^ > σ(*F*^2^) is used only for calculating *R*-factors(gt) *etc*. and is not relevant to the choice of reflections for refinement. *R*-factors based on *F*^2^ are statistically about twice as large as those based on *F*, and *R*- factors based on ALL data will be even larger.

### Fractional atomic coordinates and isotropic or equivalent isotropic displacement parameters (Å^2^)

**Table:**

	*x*	*y*	*z*	*U*_iso_*/*U*_eq_	
S1	0.77562 (5)	0.12622 (5)	0.06150 (7)	0.05989 (19)	
C1	0.51607 (18)	0.11596 (17)	0.3360 (3)	0.0561 (5)	
N2	0.60693 (16)	0.11489 (15)	0.2619 (3)	0.0597 (5)	
C11	0.70493 (19)	0.11725 (18)	0.3637 (3)	0.0586 (5)	
H11	0.7168	0.1176	0.4870	0.070*	
C9	0.97699 (19)	0.1197 (2)	0.2631 (3)	0.0658 (6)	
H9	1.0540	0.1186	0.3003	0.079*	
O3	0.23334 (13)	0.11016 (14)	0.5127 (2)	0.0722 (5)	
N1	0.95778 (19)	0.12583 (16)	−0.0612 (3)	0.0693 (5)	
C10	0.91555 (18)	0.12453 (18)	0.0913 (3)	0.0576 (5)	
C4	0.32882 (18)	0.11543 (17)	0.4617 (3)	0.0572 (5)	
O1	1.05738 (18)	0.12534 (19)	−0.0377 (3)	0.0968 (7)	
O2	0.88984 (18)	0.12695 (18)	−0.2095 (2)	0.0922 (6)	
C7	0.79851 (18)	0.11941 (18)	0.2889 (3)	0.0554 (5)	
C8	0.90855 (19)	0.1166 (2)	0.3776 (3)	0.0657 (6)	
H8	0.9353	0.1131	0.5013	0.079*	
C2	0.5164 (2)	0.1630 (2)	0.4951 (3)	0.0660 (6)	
H2	0.5804	0.1956	0.5607	0.079*	
C3	0.4248 (2)	0.1628 (2)	0.5585 (3)	0.0661 (6)	
H3	0.4273	0.1944	0.6663	0.079*	
C5	0.32604 (19)	0.0703 (2)	0.3006 (3)	0.0643 (6)	
H5	0.2614	0.0390	0.2339	0.077*	
C6	0.41788 (18)	0.0713 (2)	0.2382 (3)	0.0616 (6)	
H6	0.4144	0.0416	0.1285	0.074*	
C12	0.2326 (3)	0.1537 (3)	0.6792 (4)	0.0895 (9)	
H12A	0.1627	0.1402	0.7023	0.134*	
H12B	0.2435	0.2259	0.6754	0.134*	
H12C	0.2911	0.1245	0.7725	0.134*	

### Atomic displacement parameters (Å^2^)

**Table:**

	*U*^11^	*U*^22^	*U*^33^	*U*^12^	*U*^13^	*U*^23^
S1	0.0572 (3)	0.0756 (4)	0.0446 (3)	−0.0047 (3)	0.0102 (2)	−0.0023 (3)
C1	0.0560 (12)	0.0581 (13)	0.0533 (12)	−0.0017 (10)	0.0132 (10)	0.0017 (10)
N2	0.0581 (11)	0.0657 (12)	0.0558 (11)	−0.0027 (9)	0.0163 (9)	0.0021 (9)
C11	0.0615 (13)	0.0663 (14)	0.0499 (12)	−0.0029 (11)	0.0184 (10)	0.0034 (11)
C9	0.0530 (12)	0.0927 (18)	0.0504 (13)	−0.0001 (12)	0.0120 (10)	−0.0022 (12)
O3	0.0560 (9)	0.0878 (12)	0.0758 (11)	−0.0055 (8)	0.0235 (8)	−0.0134 (9)
N1	0.0777 (14)	0.0796 (14)	0.0574 (13)	−0.0052 (12)	0.0302 (11)	−0.0056 (11)
C10	0.0595 (12)	0.0671 (13)	0.0487 (12)	−0.0024 (11)	0.0193 (10)	−0.0034 (11)
C4	0.0505 (11)	0.0578 (13)	0.0617 (14)	0.0034 (10)	0.0129 (10)	−0.0004 (11)
O1	0.0818 (13)	0.1392 (19)	0.0829 (14)	−0.0052 (13)	0.0452 (11)	−0.0052 (13)
O2	0.1034 (14)	0.1289 (18)	0.0462 (10)	−0.0080 (13)	0.0236 (10)	−0.0058 (11)
C7	0.0579 (12)	0.0619 (13)	0.0471 (12)	−0.0052 (10)	0.0158 (9)	0.0001 (10)
C8	0.0590 (13)	0.0936 (18)	0.0433 (12)	−0.0005 (12)	0.0122 (10)	−0.0002 (12)
C2	0.0553 (13)	0.0729 (15)	0.0680 (15)	−0.0135 (11)	0.0141 (11)	−0.0142 (12)
C3	0.0628 (14)	0.0727 (15)	0.0639 (15)	−0.0098 (12)	0.0192 (12)	−0.0175 (12)
C5	0.0508 (12)	0.0765 (16)	0.0612 (15)	−0.0081 (11)	0.0082 (10)	−0.0097 (12)
C6	0.0572 (13)	0.0746 (16)	0.0500 (13)	−0.0030 (11)	0.0099 (10)	−0.0063 (11)
C12	0.0811 (18)	0.106 (2)	0.094 (2)	−0.0108 (16)	0.0447 (16)	−0.0271 (17)

### Geometric parameters (Å, º)

**Table:**

S1—C10	1.709 (2)	N1—C10	1.429 (3)
S1—C7	1.717 (2)	C4—C5	1.380 (3)
C1—C2	1.384 (3)	C4—C3	1.381 (3)
C1—C6	1.389 (3)	C7—C8	1.365 (3)
C1—N2	1.414 (3)	C8—H8	0.9300
N2—C11	1.268 (3)	C2—C3	1.372 (3)
C11—C7	1.449 (3)	C2—H2	0.9300
C11—H11	0.9300	C3—H3	0.9300
C9—C10	1.350 (3)	C5—C6	1.370 (3)
C9—C8	1.400 (3)	C5—H5	0.9300
C9—H9	0.9300	C6—H6	0.9300
O3—C4	1.366 (3)	C12—H12A	0.9600
O3—C12	1.420 (3)	C12—H12B	0.9600
N1—O1	1.214 (3)	C12—H12C	0.9600
N1—O2	1.232 (3)		
			
C10—S1—C7	89.19 (10)	C11—C7—S1	119.49 (17)
C2—C1—C6	117.6 (2)	C7—C8—C9	113.0 (2)
C2—C1—N2	124.5 (2)	C7—C8—H8	123.5
C6—C1—N2	117.8 (2)	C9—C8—H8	123.5
C11—N2—C1	119.9 (2)	C3—C2—C1	121.7 (2)
N2—C11—C7	120.3 (2)	C3—C2—H2	119.2
N2—C11—H11	119.9	C1—C2—H2	119.2
C7—C11—H11	119.9	C2—C3—C4	119.8 (2)
C10—C9—C8	110.4 (2)	C2—C3—H3	120.1
C10—C9—H9	124.8	C4—C3—H3	120.1
C8—C9—H9	124.8	C6—C5—C4	120.4 (2)
C4—O3—C12	118.2 (2)	C6—C5—H5	119.8
O1—N1—O2	124.1 (2)	C4—C5—H5	119.8
O1—N1—C10	118.6 (2)	C5—C6—C1	121.2 (2)
O2—N1—C10	117.3 (2)	C5—C6—H6	119.4
C9—C10—N1	125.7 (2)	C1—C6—H6	119.4
C9—C10—S1	114.89 (17)	O3—C12—H12A	109.5
N1—C10—S1	119.41 (18)	O3—C12—H12B	109.5
O3—C4—C5	116.0 (2)	H12A—C12—H12B	109.5
O3—C4—C3	124.7 (2)	O3—C12—H12C	109.5
C5—C4—C3	119.3 (2)	H12A—C12—H12C	109.5
C8—C7—C11	128.1 (2)	H12B—C12—H12C	109.5
C8—C7—S1	112.44 (16)		
			
C2—C1—N2—C11	30.4 (4)	C10—S1—C7—C8	0.3 (2)
C6—C1—N2—C11	−153.0 (2)	C10—S1—C7—C11	−179.7 (2)
C1—N2—C11—C7	−178.1 (2)	C11—C7—C8—C9	179.8 (2)
C8—C9—C10—N1	−178.2 (2)	S1—C7—C8—C9	−0.2 (3)
C8—C9—C10—S1	0.3 (3)	C10—C9—C8—C7	−0.1 (3)
O1—N1—C10—C9	−2.1 (4)	C6—C1—C2—C3	2.4 (4)
O2—N1—C10—C9	177.6 (3)	N2—C1—C2—C3	178.9 (2)
O1—N1—C10—S1	179.5 (2)	C1—C2—C3—C4	−0.6 (4)
O2—N1—C10—S1	−0.8 (3)	O3—C4—C3—C2	179.6 (2)
C7—S1—C10—C9	−0.4 (2)	C5—C4—C3—C2	−1.0 (4)
C7—S1—C10—N1	178.2 (2)	O3—C4—C5—C6	−179.8 (2)
C12—O3—C4—C5	178.8 (2)	C3—C4—C5—C6	0.8 (4)
C12—O3—C4—C3	−1.8 (4)	C4—C5—C6—C1	1.0 (4)
N2—C11—C7—C8	−176.2 (2)	C2—C1—C6—C5	−2.6 (4)
N2—C11—C7—S1	3.8 (3)	N2—C1—C6—C5	−179.4 (2)

### Hydrogen-bond geometry (Å, º)

**Table:**

*D*—H···*A*	*D*—H	H···*A*	*D*···*A*	*D*—H···*A*
C8—H8···O2^i^	0.93	2.48	3.292 (3)	146
C9—H9···O3^ii^	0.93	2.40	3.273 (3)	156

Symmetry codes: (i) *x*, *y*, *z*+1; (ii) *x*+1, *y*, *z*.
